# Multiple Interacting Factors Influence Adherence, and Outcomes Associated with Surgical Safety Checklists: A Qualitative Study

**DOI:** 10.1371/journal.pone.0108585

**Published:** 2014-09-26

**Authors:** Anna R. Gagliardi, Sharon E. Straus, Kaveh G. Shojania, David R. Urbach

**Affiliations:** 1 University Health Network, Toronto, Ontario, Canada; 2 St. Michael’s Hospital, Toronto, Ontario, Canada; 3 Sunnybrook Health Sciences Centre, Toronto, Ontario, Canada; 4 University Health Network, Toronto, Ontario, Canada; University of Geneva, Switzerland

## Abstract

**Objectives:**

The surgical safety checklist (SSC) is meant to enhance patient safety but studies of its impact conflict. This study explored factors that influenced SSC adherence to suggest how its impact could be optimized.

**Methods:**

Participants were recruited purposively by profession, region, hospital type and time using the SSC. They were asked to describe how the SSC was adopted, associated challenges, perceived impact, and suggestions for improving its use. Grounded theory and thematic analysis were used to collect and analyse data. Findings were interpreted using an implementation fidelity conceptual framework.

**Results:**

Fifty-one participants were interviewed (29 nurses, 13 surgeons, 9 anaesthetists; 18 small, 14 large and 19 teaching hospitals; 8 regions; 31 had used the SC for ≤12 months, 20 for 13+ months). The SSC was inconsistently reviewed, and often inaccurately documented as complete. Adherence was influenced by multiple issues. Extensive modification to accommodate existing practice patterns eliminated essential interaction at key time points to discuss patient management. Staff were often absent or not paying attention. They did not feel it was relevant to their work given limited evidence of its effectiveness, and because they were not engaged in its implementation. Organizations provided little support for implementation, training, monitoring and feedback, which are needed to overcome these, and other individual and team factors that challenged SSC adherence. Responses were similar across participants with different characteristics.

**Conclusions:**

Multiple processes and factors influenced SSC adherence. This may explain why, in studies evaluating SSC impact, outcomes were variable. Recommendations included continuing education, time for pilot-testing, and engaging all staff in SSC review. Others may use the implementation fidelity framework to plan SSC implementation or evaluate SSC adherence. Further research is needed to establish which SSC components can be modified without compromising its effectiveness.

## Introduction

Checklists have become a commonly used tool to facilitate the delivery of high quality health care [1]. They are meant to improve patient outcomes by specifying care recommendations and health professional roles to standardize and coordinate care delivery, and enhance inter-professional communication [2], [3]. Checklists have been widely implemented in operating rooms to address preventable adverse events that arise during, or as a result of surgical procedures following publication of an international study sponsored by the World Health Organization (WHO) that showed it reduced surgical mortality and complications [4]–[6]. The surgical safety checklist (SSC) prompts oral confirmation by surgical team members of key processes at three time points – before anesthesia administration (i.e. patient identity confirmed, site marked, anesthesia safety check completed, other patient risks such as allergy, aspiration risk, and anticipated blood loss), immediately prior to incision (i.e. team member introductions, confirmation of patient, site, side and procedure being performed, discussion of any other concerns), and before the patient is taken out of the operating room (i.e. procedure recorded, instrument count, key recovery or management concerns) [6].

While the findings of several observational studies and systematic reviews support its effectiveness [7]–[9], other studies have reported inconsistent, or little to no impact of the SSC compared with usual care [10]–[12]. Variable adherence with the SSC may account for its inconsistent impact. A systematic review of 20 studies that examined the impact of the SSC on teamwork in the operating room found that hospitals which achieved higher adherence with SSC use were more likely to significantly reduce postoperative complications [13]. However, multiple studies demonstrated that recorded adherence is unreliable and significantly higher than observed adherence [14]–[16].

Several processes and factors may influence adherence with the SSC and, hence it’s capacity to improve surgical outcomes. Adaptation, either modification or removal of one or more of the processes at any of the three time points specified in the SSC, may limit team interaction, the completion of crucial tasks, or the delivery of essential components of care [17]. Implementation may not be successful if no interventions are used to promote use of the SSC, or if those strategies are less than effective. For example, some research suggests that SSC use must be aligned with institutional values, enabled with training and coaching, and encouraged by local champions and real-time feedback of performance data [7], [8]. Poor or incomplete integration of the SSC into operating room processes may limit adherence if staff are not aware of it, or can easily bypass its use in favour of usual practices [7], [8], [18]. Monitoring may be necessary to identify and address adherence issues and ensure sustained SSC use [17], [19]. Adherence may also be influenced by individual (beliefs about SSC effectiveness, uncertainty about when and how to use it, perceived interruption of workflow and redundancy with other checklists or processes, resistance to change) and team-related factors (professional hierarchy in the operating room where individual physicians rather than multidisciplinary teams make decisions) [8], [9], [13], [20]–[23].

A large proportion of in-hospital adverse events are surgery-related and preventable [24]. The SSC may prevent such adverse events but its impact has been inconsistent due to variable adherence. Research to date has not revealed how to promote and support SSC adherence. One, few qualitative studies explored SSC introduction and use so we have little insight on whether and how various processes and factors such as those described above may influence adherence. Two, most of those studies involved one or a few participating hospitals so findings may be setting-specific. Three, adoption of an innovation is not a discrete event and must be evaluated longitudinally [25]. Most studies assessed adherence or clinical impact from one week to three months after the SSC was introduced so adoption may not yet have occurred, and issues influencing adherence may not yet have arisen. The overall objective of this study was to understand and compare the processes and factors influencing SSC adherence in multiple hospitals of different types that had been exposed to the SSC for various lengths of time. In particular, we interviewed nurses, surgeons and anaesthetists from community and academic hospitals in several jurisdictions that had used the SSC for one year or less, or longer than one year to learn how various processes and factors influenced SSC adherence. This may reveal approaches for optimizing SSC adherence, which could be tested in future research and, if widely adopted, lead to more consistent SSC use, and improved outcomes among surgical patients.

## Methods

### Approach

Qualitative interviews were conducted to explore how adaptation, implementation, integration and monitoring or other process, and individual, team or other factors influenced SSC adherence. Qualitative research elicits detailed information about beliefs and experiences, and the factors that shape them to create a thorough understanding of an issue. A grounded theory approach was used to collect and analyze data such that views, experiences, needs and suggestions emerged freely during interviews and inductively during data analysis rather than being restricted to the components of established theory [26]. Rigour was optimized by sampling participants with various characteristics that could influence their views and experiences; exploring responses inductively for emerging ideas; demonstrating responses from an array of participants by anonymously identifying exemplary quotes; comparison of independently-derived analysis across two individuals, and thorough, high-level interpretation of the findings [27]. It was further ensured by complying with Relevance, Appropriateness, Transparency and Soundness (RATS) principles for reporting of qualitative research [28]. This research was based in Canada which features ten provinces and three territories, and where the health care system is largely publicly funded. This research was approved by the University Health Network Research Ethics Board (10-0659-AE) and all participants signed a consent form prior to interviews.

### Sampling and recruitment

Purposive sampling was used to identify practicing clinicians with differing characteristics including role (nurses, surgeons, anaesthetists), geographic region (provinces), type of hospital (teaching, large community, small community less than 100 beds) and time using the SSC (≤12 months, 13+ months). Hospitals that had adopted the surgical checklist were identified by the Canadian Patient Safety Institute (http://www.patientsafetyinstitute.ca/) which advocated for SSC adoption in Canada. The list of hospitals they provided included the name and contact information for a front line nurse or nurse leader who was most familiar with implementation of the SSC at each hospital. Contact information for surgeons and anesthetists at those hospitals was acquired through random sampling of publicly available directories from the College of Physicians and Surgeons for respective provinces. All individuals were invited to participate by regular mail or email with an invitation letter and consent form. A reminder was sent to non-respondents at two and four weeks from initial contact. The intent was to recruit, from among those who consented, 10 of each professional role from across Canada who differed in non-mutually exclusive fashion by type of hospital and time using the SSC for a minimum total of 30 participants.

### Data collection

Interviews were conducted in English language with all consenting participants via telephone by a trained research assistant. Participants were asked about perceived benefits of, and adherence with the SSC; how the SSC was adapted, implemented, integrated and monitored; factors influencing these processes; and suggestions for improving SSC adherence. Interviews of approximately 45 minutes were audio-recorded, then transcribed verbatim. Interviews were conducted over eight months from January 6, 2011 to August 14, 2011. Detailed information from representative, rather than a large number of cases is needed in qualitative research [26]. Sampling was concurrent with data collection and analysis, and proceeded until unique themes no longer emerged from successive interviews (saturation). This was determined by discussion of emerging themes between two independent reviewers, the principal investigator and research assistant.

### Data analysis

Unique themes were identified in an inductive manner through iterative stages [29], [30]. First, interview transcripts were read to identify, define and organize themes in participant responses relevant to each of the main interview questions (first level coding). Second, a codebook was developed to organize codes reflecting emerging themes, their definition, sample quotes illustrating application of that code, and an account of decisions related to that code. Third, transcripts were reviewed (constant comparative technique) to assess whether and how to expand or merge themes (second level coding). Interview transcripts and the codebook were analyzed independently by the research analyst and principal investigator. The two met to compare findings and achieve consensus by discussion. Data (quotes labeled with an anonymous identifier reflecting profession, province, type of hospital and time using the SSC) were tabulated for each main interview question by theme and profession to identify trends.

Once data were analyzed and organized in this manner, the findings were further interpreted according to a conceptual framework of implementation fidelity, defined as the degree to which programs are implemented as intended [31]. Implementation fidelity must be evaluated to assess whether lack of impact of a program is due to poor implementation or inadequacies in the program. This framework proposes that program Adherence (program is delivered or used as intended) is influenced by several inter-related factors including program Differentiation (core elements essential for success are known and maintained), Facilitation (training, tools, interventions to promote and support adherence), Quality of delivery (whether and how participants undertake or use the program in an ideal manner), participant Responsiveness (participants are engaged in, and view the program as relevant) and Complexity (number and type of issues challenging adherence). To do this, themes and exemplary quotes from interview findings were matched with domains of the framework. For example, themes related to adherence and adaptation corresponded directly with Adherence and Differentiation, respectively. The framework domain Responsiveness was populated with themes about perceived benefits of the SSC, and themes about engagement and relevance within interview findings related to SSC implementation. The framework domain of Quality was populated with themes within interview findings related to SSC integration. The framework domains of Facilitation and Complexity were populated with themes reflecting organizational, team and individual factors from within findings relevant to implementation, integration and monitoring.

## Results

### Participants

Of 607 physicians invited to participate, 551 either declined or did not respond, 56 consented and 22 were interviewed. Of 306 nurses invited to participate, 226 either declined or did not respond, 80 consented and 29 were interviewed. Participants included 29 nurses, 13 surgeons and 9 anaesthetists from 18, 14 and 29 small community, large community and teaching hospitals, respectively (Table 1). Thirty-one had used the SSC for 12 months or less, and 20 had used the SSC for 13 or more months. Participants represented the provinces of British Columbia (10), Alberta (10), Saskatchewan (1), Ontario (17), Quebec (3), New Brunswick (6), Prince Edward Island (1), and Nova Scotia (3).

**Table 1:** Participant characteristics by profession, type of hospital and time using the surgical checklist.

Type of Hospital	Profession			Subtotal
	Nurse	Surgeon	Anesthetist	
Small community (<100 beds)	14	3	1	18
≤12 mo	11	3	–	14
13+ mo	3	–	1	4
Large community	7	3	4	14
≤12 mo	4	2	4	10
13+ mo	3	1	–	4
Teaching	8	7	4	19
≤12 mo	3	3	1	7
13+ mo	5	4	3	12
Subtotal	29	13	9	51

### SSC adherence

Study findings organized by main interview question, then theme and type of health professional are available in Table S1 (Adherence with Surgical Safety Checklists: Summary of study findings). When asked about adherence, most participants said that the SSC was incompletely and inconsistently reviewed and documented for each patient. Several mentioned that review of the SSC was marked as complete even when this was not the case. Experiences or views were similar across participants from different geographic regions, and from different health professional groups. Some differences by hospital type and length of time using the SSC were apparent. They are noted in the following interpretation of study findings according to the domains of the implementation fidelity conceptual framework, which was used to organize the multiple processes and factors that influenced adherence. The interpretation is summarized in Table 2 with select participant quotes.

**Table 2:** Key findings according to a conceptual framework of implementation fidelity.

Framework Domain	Themes from Study Findings	Exemplary Quotes from Study Findings
Adherence (SSC used - all items reviewed by team at key times and documented)	Portions not completed for every patient	No one goes through the whole list of every single item (Sx01ON-T-short); Consistent application of the checklist in every case (Ax09NB-T-long); They trickle out with the debriefing (Nx05ON-S-short); Getting the surgeons to be in the room for the briefing (Nx03AB-L-long)
	Documentation incomplete or inaccurate	If some part of the checklist were done then it’s considered complete (Sx04BC-T-long); Nurses are biased to mark it complete otherwise the manager will say how come this case went ahead (Sx11ON-T-long); There is no recording that each component was asked or that each individual was present (Sx09ON-T-long)
Differentiation (Core elements essential for success are maintained)	Adapted other versions or newly developed	We came up with our own (Nx06NS-L-short); As issues came up there would be discussions about what should or should not be in the checklist (Sx09ON-T-long)
	Modification viewed as necessary	It needs to be easily modifiable (Sx11ON-T-long); Flexibility in modification of the checklist (Sx07AB-L-short)
Responsiveness(Participants are engaged and view the program as relevant)	Numerous perceived benefits were noted	It builds and fosters a team mentality (Sx04BC-T-long); It reassures the family (Sx06AB-T-short); We’ve improved the use of appropriate peri-operative antibiotics (Ax06NB-T-long); Mislabeling of specimens is really improved (Nx15BC-S-short)
	In contrast, use was imposed so staff did not feel engaged, and SSC relevance was questioned	It’s been forced on us. It’s hurt morale and caused tension in the OR (Ax05BC-L-short); Involve the people who’re gonna be using it in its development so you feel ownership (Sx05BC-S-short); It’s an additional layer that doesn’t improve efficiency (Ax07BC-L-short); Lingering beliefs in why it is necessary (Sx07AB-L-short)
Quality (Participants use the SSC in an ideal manner)	Staff absent at key times, not paying attention or obstructive	Surgeons were refusing to do it (Sx04BC-T-long); Some days it doesn’t seem like a team effort (Ax09NB-T-long); Everybody being in the room at the same time was one of our biggest challenges (Nx09ON-S-short)
	SSC reviewed but not in a mindful manner	There are a lot of people that are just going through the motions (Sx12BC-T-long); People who aren’t listening, aren’t participating, rolling their eyes (Nx01NB-L-short)
	Available in OR as card or wall poster	Small cards were placed in every OR (Ax07BC-L-short); We have a large poster in each of the ORs (Sx08BC-T-short)
	Documentation processes variable	Nurses in the OR have a booklet where they mark off for every case if the surgical checklist was used (Nx10AB-S-short); Nurses have three check boxes on the electronic patient record (Ax01ON-T-long)
Facilitation (Resources, training, interventions or other strategies provided or enabled by the organization that promoted and supported adherence)	Implemented by alerting staff to impending use	We were informed by letters (Ax08NB-L-short); A memo went out to everybody that it was gonna be starting (Nx05NS-L-short)
	Little time to prepare or pilot-test	We would probably start one service at a time because it was extremely overwhelming to implement it all at the same time in five services (Nx20SK-S-long)
	Little training on SC use	We don’t know how to do it, we’re not trained (Sx12BC-T-long); They could have done a better job of educating people (Ax04NB-T-short)
	Little support from facilitators	Identify surgeon champions (Sx04BC-T-long); Need leaders in the physician group to sell it (Nx23ON-T-long)
	Little support from hospital leadership (resources or visible involvement)	Needs to be supported by the medical quality committee of the hospital (Ax02ON-L-short); I just didn’t get the support that I would have needed to be able to implement that fully (Nx02ON-L-long)
	Monitoring and feedback varied	We do audits quarterly (Nx01NB-L-short); Feedback that was accurate and meaningful (Ax07BC-L-short)
	Non-adherence addressed at few sites	I follow up with them individually (Nx12ON-S-short); We try to review it in the OR committee monthly meeting (Sx06AB-T-short)
	No incentives or accountability	There are no consequences (Nx02ON-L-long); There’s no punishment (Sx07AB-L-short); If it was mandatory people might be more diligent (Nx05NS-L-short)
Complexity (Number and type of issues challenging adherence - team and individual factors)	Beliefs about effectiveness	Ongoing data demonstrating its impact on patient care (Sx09ON-T-long); People like to know this is evidence-based (Nx29AB-T-long); There was little evidence that it has been proven to make any difference in patient outcome (Ax05BC-L-short)
	Resistance to change	Getting acceptance from surgeons and anaesthesiologists (Ax07BC-L-short); Older practitioners are very set in their ways and it’s hard to change them (Sx11ON-T-long)
	Concerns about delays	It does delay getting the case started (Sx04BC-T-long); Cause of inefficiency in our operating room (Ax05BC-L-short)
	Lack of knowledge about how to use it	People were not fully understanding what the components of the checklist were (Nx25ON-T-long); I’m still unclear as to who it is that’s supposed to lead the discussion (Sx04BC-T-long)
	Increased workload for nurses	It’s one more job for the nurse to do (Nx02ON-L-long); Layering on further administrative burden (Ax07BC-L-short); Documentation has dramatically increased, and it has taken away from patient care (Sx09ON-T-long)
	Hierarchical OR culture is a barrier to team interaction	Nurses were intimidated so there was friction between team members (Ax02ON-L-short); Flatten the hierarchy (Sx12BC-T-long); Some nurses are really shy to speak up (Nx26ON-T-short)

Profession (Nx = nurse, Sx = surgeon, Ax = anesthetist), Province (two-letter identifier), Hospital type (S = small, L = large, T = teaching), Time using the surgical checklist (short = ≤12 months, long = 13+ months).

### Factors influencing SSC adherence

#### Differentiation

Most participants said that they modified the SSC considerably. In several cases one or more versions were blended to create an entirely new SSC. The SSC was often modified to make it easier to use and accommodate existing local practices or processes, for example, who should be present for completion of each component, and where components were completed if not in the operating room. Therefore multidisciplinary interaction at key time points may not have occurred. Participants who had used the SSC for longer periods of time were more likely to say that they had added dimensions to the checklist (i.e. handoffs, anesthesia relevant items, patient positioning and destination following surgery), changed the order of items, and had adapted it over an extended period of time. Participants from small hospitals were more likely to remove items relevant to surgical procedures that they did not perform, and waive the need for team introductions. Rapid turnovers, call backs and emergency cases also challenged SSC adherence. Modification was viewed as necessary, so maintaining the integrity of the SSC was not a concern. In fact, a few physicians recommended that the SSC should be more easily modifiable to suit different procedures.

#### Responsiveness

Most participants viewed the SSC as relevant and cited numerous benefits associated with its use. This included better use of time in the operating room; improved communication, teamwork and staff satisfaction; and patients and families were comforted knowing that the SSC was used. Several said that, due to the SSC, they were better prepared including having the proper equipment and antibiotics available, consistent site marking, correct labelling of specimens, and informed consent for all procedures. In general this enabled the right surgery on the right patient on the right side. A few noted that incidents had been averted. Despite these positive remarks, engagement was viewed as less than ideal. For example, many participants said that staff should have been involved in adapting and implementing the SSC as a means of fostering ownership. Instead they felt it had been imposed on them by management, which hurt morale and caused tension in the operating room. They also questioned why it was necessary and doubted that it improved efficiency, therefore it was not perceived as highly relevant.

#### Quality

The SSC was available in operating rooms as a laminated card or sheet, or wall poster, and verbally reviewed as was the intent. However, the intended multidisciplinary interaction among staff was frequently not achieved. Staff were often absent at one or more of the three key time points. Some were even obstructive by refusing to participate in SSC review. Even when SSC items were being reviewed, staff were talking or not paying attention, and this was described as “going through the motions”, which diminished the impact of the SSC to “just another tick box”. Therefore the quality of inter-professional communication was limited, and items pertinent to patient safety may not have been reviewed in a mindful manner. Processes in place to document that the SSC had been reviewed were variable across hospitals. In some sites this was done verbally only. Other hospitals used either paper forms or electronic medical records to document completion of either the entire SSC or each of its three components. Those using the SSC for a shorter period of time were not aware if completion was documented for each patient.

#### Facilitation

Little organizational support was available to promote and facilitate SSC use. Staff were informed by email or memo that they were to use the SSC. Nurses, who were largely responsible for planning SSC implementation, said that there was little time to prepare or pilot-test the SSC prior to full launch, and that no resources were provided by hospital management for more thorough or robust implementation efforts. Furthermore, hospital leadership was not seen as involved in either promoting or actively implementing the SSC. Many participants said that they did not receive information or training on how to use the SSC, and that identifying facilitators, in particular physician champions, would have improved SSC uptake.

Monitoring of SSC use was variable. Some participants, largely from hospitals using it for a shorter length of time, said that audit reports based on nursing documentation of SSC use were shared with staff and hospital managers but this was weekly at some sites and yearly at others. A few sites monitored SSC use with observational spot checks. There appeared to be no incentives to prompt SSC use or consequences associated with non-adherence. Some nurse managers held discussions with non-compliant individuals. Those using the SSC for a longer period of time were more likely to say that adherence issues resulted in an incident report and/or discussion among the operating room team or surgical services committee. Rarely was non-adherence reported to those in leadership roles. Several participants recommended regular local audits of SSC use, or random audits conducted by groups independent of the operating room team or external to the organization, and consequences associated with non-compliance. In contrast, others said that SSC use would be promoted if local data were more routinely collected, analyzed and shared to demonstrate its impact, necessitating additional clerical support to alleviate the nursing workload.

#### Complexity

Multiple individual and team factors interacted to influence adherence. All participants noted that the nursing workload had increased, in large part due to the need for SSC documentation. Many participants said that staff were uncertain about how to use the SSC and who was responsible for leading it. Participants said that SSC use was limited by the traditionally physician-dominated hierarchical culture of the operating room and lack of confidence among nurses leading SSC review, particularly when faced with resistance from staff who were most often surgeons, leading to tension and avoidance of SSC review. Physician resistance to change was attributed to individual beliefs about relevance given the lack of strong evidence on SSC effectiveness, physician age, perceived redundancy with other checklists or processes, and concerns about surgical delays as a result of taking time to review the SSC. To rectify these issues, participants recommended that surgeons lead review of the SSC, continuing education be offered on how to use the SSC, and networking take place with other hospitals to share strategies for promoting SSC compliance.

#### Interpretation Summary

Use of the implementation fidelity conceptual framework to analyze study findings confirms that multiple processes and factors influence SSC adherence, including issues that were proposed in other research and several unique factors that emerged from this research. Staff did not adhere to the technical and qualitative manner in which the SSC was meant to be used. In part this was because they did not feel it was relevant to their work, and had not been engaged in its adaptation and implementation, so did not fully or meaningfully take part in reviewing the SSC for each patient (Responsiveness, Quality - qualitative manner). In part this was due to the fact that extensive modification of the SSC to accommodate existing, preferred practice patterns eliminated essential interaction to discuss patient management issues at key time points (Differentiation - technical manner). Complexity of the SSC was high given the many individual and team factors that further challenged SSC adherence. Organizations provided few resources and supports for thorough implementation, training, monitoring and feedback (Facilitation), which are clearly needed to overcome challenges associated with Responsiveness, Differentiation, Quality and Complexity. It is notable that, overall, responses were similar across different types of health professionals and hospitals, and among those exposed to the SSC for different periods of time.

## Discussion

This study found that SSC adherence was poor though often documented as complete, as was identified in other studies [7], [14]–[16]. This was at odds with many positive views articulated about its benefits. Analysis of findings using an implementation fidelity conceptual framework revealed that multiple processes and factors influenced SSC adherence. This may explain why, in studies evaluating SSC impact, outcomes were variable despite documentation of high adherence. This study confirms the assertion by Carroll et al. that any evaluation of adherence with a quality improvement intervention such as the SSC must measure not only adherence, but all of the implementation fidelity domains which influence it, and that evaluation must be longitudinal since issues may not emerge immediately upon introduction of the intervention [31]. This would also suggest that those planning to introduce the SSC or other quality improvement intervention could use the implementation fidelity conceptual framework as a means of planning how to promote, support and sustain its use.

The SSC was adapted to accommodate existing practice patterns. Therefore the intended timing and quality of team interaction at key intervals was not achieved. No one questioned SSC integrity, likely because tailoring was encouraged by national and international SSC advocates [6]. Further research is needed on the extent to which SSC modification influences adherence and associated clinical outcomes. This would establish which elements must remain in the SSC, and which can be modified and how without compromising effectiveness. This too is recommended by Carroll et al. who state that an evaluation of outcomes “…may identify those components that are essential to the intervention, and must be implemented if the intervention is to have its intended effects. This evaluation may in turn inform the content of the intervention by determining the minimum requirements for high implementation fidelity” [31].

Use of the SSC was mandated by government and hospital management, and email, sometimes supplemented with meetings, was widely used to notify staff about the SSC. Educational material and meetings are known to have minimal impact on the uptake of innovations [32]. Participants recommended staff engagement, multidisciplinary interaction, use of champions and facilitators, and more time to thoughtfully plan, develop, pilot-test and implement the SSC. Those using the SSC for a longer period of time reflected on the lack of resources provided by management to support SSC implementation, and of advocacy from professional societies. Leadership promotion of, and involvement in strategic initiatives are recognized as determinants of success and organizational performance [33]–[35]. Participants also recommended ongoing networking with other hospitals to learn from each other’s experiences. Knowledge networks, quality improvement collaboratives and communities of practice are known to support collaboration and the exchange of knowledge to achieve a common goal in both the management and health care sectors [36]–[38]. Tools exist for diagnosing barriers or organizational quality improvement culture [39]–[41]. This would establish baseline levels of acceptance and capacity to inform the selection of one or more implementation strategies appropriate for overcoming identified challenges.

The SSC was widely available in operating rooms in the form of a laminated card or wall poster. However portions were often not completed, and staff were absent or not paying attention. Those using the SSC for a shorter time noted uncertainty about how to use it. Even among those who used it for a longer period of time, physician resistance was evident and, given traditional operating room hierarchy, nurses were reluctant to direct physicians. In many cases it was marked as having been completed despite these circumstances. These issues were also revealed in other research [8], [9], [20]–[23]. To address resistance participants recommended that physicians assume the responsibility for leading SSC review. This was evaluated in a study where each team member was required to lead the review of the SSC component most relevant to their role [42]. Completion of all items increased from 54% when previously surgeon-led to 97% after the intervention, and the improvement was sustained at 18 months. Social identity theory refers to shared knowledge, values and practices in members of social or professional groups, however social identity can be threatened when individuals are forced to collaborate with members of other groups, therefore continuing education on SSC use must be multidisciplinary in nature [43]. Such an educational program could also include conflict management training that describes, and provides alternatives for negative behaviour [44].

Sharing of audit data with staff appeared more common among those using the SSC for a shorter period of time. Those using the SSC for a longer period of time were more likely to have team conversations about adherence. Otherwise there were no repercussions for non-compliance. Perhaps this was due to resolution through discussion, or perhaps because no accountability mechanisms were in place to address non-compliance. Some participants recommended that SSC use should be mandatory; monitored with regular or random audits by an arms-length group; and that consequences be applied for non-compliance. Monitoring and sharing of performance data with staff, and consequences such as reprimanding and penalizing were found to improve SSC compliance in other research [17], [19]. However, evidence on the impact of punitive, top-down strategies to motivate performance is mixed, and conflicts with theories of adult learning [45], [46]. In contrast, others said that external research or local data demonstrating impact would promote SSC use. While this would require clerical support to alleviate the nursing workload, audit and feedback can improve professional practice, particularly when the source is a supervisor or colleague, it is provided more than once, it is delivered in both verbal and written formats, and when it includes both explicit targets and an action plan [47].

Transferrability of our finding to other settings may be limited. We attempted to mitigate this through purposive sampling of participants based on a range of characteristics that may have influenced their views. Those using the SSC for a longer period of time noted that they had adapted it over an extended period of time, and non-compliance was addressed through team discussion. This suggests that the process of adoption is not linear, and time may be needed to use the SSC, experience challenges, work out solutions, develop teamwork, monitor adherence, and address ongoing non-compliance. However, most challenges and recommendations were remarkably similar across participants representing different regions, professions, types of hospitals and length of exposure to the SSC, and confirm previous research. Therefore these findings may be broadly relevant. Applicability of the findings may be limited because data were collected in 2011. However, impact of the surgical checklist remains topical given inconsistent findings across studies, and the findings offer insight on how to enhance SSC adherence. Some researchers favor in-person interviews to collect non-verbal cues that can be used to further interpret meaning while others prefer telephone interviews because participants may often be more forthcoming when not faced by the interviewer [26]. We chose the latter approach, in part because telephone interviews can be easily accommodated in busy professional schedules, and minimize research costs when participants are geographically dispersed as in this study. Data suggest that participants were quite frank about their experiences thus having conducted telephone interviews may not have limited our data collection.

Overall, this study supports the assertion that variability in SSC impact across previous studies may be attributed to multiple processes related to adapting, implementing, integrating and monitoring SSC use, and interacting organizational, team and individual factors that influenced SSC adherence. Views were mixed about accountability, so further research is needed to identify the approaches that best incentivize SSC adherence. Some participants noted that evidence of SSC impact is needed to convince clinicians of its importance and motivate adherence. Concerns about effectiveness were also identified in other studies of SSC use [8], [9]. The need for longitudinal evaluation of outcomes is also evident. It would be interesting to repeat interviews among the same population to explore whether adherence had improved and why, or whether it was challenged by the same or different factors. Future studies to demonstrate SSC impact should aim to better implement and integrate the SSC, and assess its impact only after a sufficient period of adjustment. Planning and evaluation could be guided by the domains of the implementation fidelity conceptual framework, which proved to be very useful in analyzing the myriad of contextual factors influencing SSC adherence.

## Supporting Information

Table S1 Adherence with Surgical Safety Checklists: Summary of study findings.
(DOC)
