# Supplementary material for: Multiple Interacting Factors Influence Adherence, and Outcomes Associated with Surgical Safety Checklists: A Qualitative Study
Source: PLoS One. 2014 Sep 26;9(9):e108585. doi: 10.1371/journal.pone.0108585 (PMC4178177; doi:10.1371/journal.pone.0108585)
Supplement: Table S1 — Adherence with Surgical Safety Checklists: Summary of study findings. (DOC) [file pone.0108585.s001.doc]

Adherence with the surgical safety checklist

| Theme | Exemplary quotes from participants | | |
| --- | --- | --- | --- |
|  | Nurses | Surgeons | Anaesthetists |
| Portions of the SSC not completed for every patient | - Debrief is our least successful component (Nx22AB-T-long) | - No one goes through the whole list of every single item. It varies from surgeon to surgeon and what they think is important (Sx01ON-T-short) | - Inconsistent application of the checklist in every case (Ax09NB-T-long) |
| Documentation incomplete, or inaccurate | - I’ve never gone back and did an audit whether it’s on every chart or not (Nx07NS-L-short) | - If some part of the checklist were done then it’s considered complete (Sx04BC-T-long) - Nurses mark it complete or the manager will ask why this case went ahead (Sx11ON-T-long) | - No it does not get recorded (Ax08NB-L-short) |

Profession (Nx=nurse, Sx=surgeon, Ax=anesthetist), Province (two-letter identifier), Hospital type (S=small, L=large, T=teaching), Time using the surgical checklist (short=≤12 months, long=13+ months)

**Perceived benefits of the surgical safety checklist**

| Theme | Exemplary quotes from participants | | |
| --- | --- | --- | --- |
|  | Nurses | Surgeons | Anaesthetists |
| Patient safety | - Providing safer care for our patients (Nx20SK-S-long) | - Improve patient safety (Sx08BC-T-short) | - It’s safer for patients (Ax06NB-T-long) |
| Patient confidence | - People know that they have a safe lodging when they come here (Nx04PEI-L-long) | - It instils confidence in the families (Sx06AB-T-short) | --- |
| Promotes mindfulness or preparedness | - Makes people more cognizant – you’re actually really thinking about what you’re doing (Nx02ON-L-long) | - Makes sure that everyone is doing the right thing and is aware of their role (Sx08BC-T-short) | - People have a formal way in which they anticipate problems (Ax03QC-S-long) |
| Prevention of incidents and improved care | - We have evidence of catches (Nx05NS-L-short) | - We had consent for the one procedure and not the other and that was caught because of the checklist (Sx01ON-T-short) | - We prevented the wrong patient in the wrong room (Ax01ON-T-long) |
| Improved time management and efficiency | - More accurate start of our surgical time (Nx27QC-T-short) | - Speeds up the operation (Sx08BC-T-short) | - They found that it didn’t add time, that cases still flowed (Ax06NB-T-long) |
| Teamwork and communication | - It brought the team together (Nx25ON-T-long) | - Better teamwork, better spirit, better morale (Sx01ON-T-short) | - Improved communication in the operating room, improved team building (Ax06NB-T-long) |
| Staff satisfaction | - Increased staff satisfaction, decreased anxiety (Nx29AB-T-long) | --- | --- |

Profession (Nx=nurse, Sx=surgeon, Ax=anesthetist), Province (two-letter identifier), Hospital type (S=small, L=large, T=teaching), Time using the surgical checklist (short=≤12 months, long=13+ months)

**Adaptation of the surgical safety checklist**

| Theme | Exemplary quotes from participants | | |
| --- | --- | --- | --- |
|  | Nurses | Surgeons | Anaesthetists |
| Minor modifications | - We just changed it a little wee bit (Nx19QC-S-short) | - We made some minor modifications to it (Sx07AB-L-short) | - The format and structure is pretty much the same, just some of the wording (Ax-07BC-L-short) |
| Considerable modifications or creation of new checklist | - We developed it ourselves (Nx15BC-S-short) - We’ve basically done our own (Nx04PEI-L-long) - We came up with our own (Nx06NS-L-short) - We created our own (Nx25ON-T-long) | - It was developed in house based on both of those (Sx08BC-T-short) - We’ve modified it significantly (Sx09ON-T-long) | - We got the surgical checklist and took one or two things off that and put it into our own checklist (Ax02ON-L-short) |
| Adapted to surgical procedures | - We took out a couple of those things because we can’t do them here (Nx12ON-S-short) - If we don’t do those procedures we took them (Nx20SK-S-long) | - We incorporated relevant patient data and relevant information with regards to the conduct of the operation (Sx12BC-T-long) | - We adapted it because we’re an ambulatory facility (Ax01ON-T-long) - We’ve taken out parts that aren’t relevant (Ax04NB-T-short) |
| Adapted to accommodate local care processes | - The team doesn’t introduce itself each time because we’re a small hospital and know each other (Nx20SK-S-long) - For the briefing part anaesthesia and nurses are present (Nx02ON-L-long) - In the sign in area we do not have surgeons present (Nx01NB-L-short) | --- | --- |
| Adapted for ease of use and relevance | - It was shortened. Checklists out there were getting quite complicated (Nx15BC-S-short) - We needed to make it a very user friendly tool, not a bunch of questions that needed to be asked (Nx01NB-L-short) | - It needs to be a functional checklist that is relevant to the service (Sx09ON-T-long) - Parts of the checklist are not really relevant so people don’t see the value (Sx11ON-T-long) | - The perception of redundance is huge. It’s an additional layer that doesn’t improve efficiency. Should be very concise checklist of items that people can glance at and know what they’re doing (Ax07BC-L-short) |
| Adaptation over time based on assessment of use or impact | --- | - Once it had been adopted, as issues came up there would be discussions about what should or should not be in the checklist (Sx09ON-T-long) - We’ve progressively modified it (Sx12BC-T-long) | --- |

Profession (Nx=nurse, Sx=surgeon, Ax=anesthetist), Province (two-letter identifier), Hospital type (S=small, L=large, T=teaching), Time using the surgical checklist (short=≤12 months, long=13+ months)

**Implementation of the surgical safety** checklist

| Theme | Exemplary quotes from participants | | |
| --- | --- | --- | --- |
|  | Nurses | Surgeons | Anaesthetists |
| Choice of implementation strategy based on strategies used by others | - We used a package that they sent us just for implementation (Nx11BC-S-long) - We connected with counterparts across the country and asked what worked well, what didn’t (Nx04PEI-L-long) - I went to a conference and brought this back (Nx12QC-S-short) - I downloaded the guidelines from the WHO web site (Nx07NS-L-short) - We sort of stumbled across the strategy, not necessarily based on an educated choosing of the strategy (Nx20SK-S-long) - I searched different places for information and then I came up with something (Nx27QC-T-short) | - We just followed what [hospital] did in their implementation (Sx11ON-T-long) - We accessed information that was available online (Sx06AB-T-short) - We went for a site visit to [hospital] where there is a physician who was part of the provincial team and then we invited him for rounds (Sx11ON-T-long) | --- |
| Email, letter, handouts or informational posters | - A memo went out to everybody that it was gonna be starting and we wanted everyone’s participation (Nx05NS-L-short) - We put handouts in physician boxes (Nx17AB-S-short) - Articles were posted (Nx04PEI-L-long) - We put up posters around the department and said that as of this date we were going to be trialing a new surgical safety checklist (Nx14ON-S-short) | - Memo via email sent out by the department head (Sx07AB-L-short) - Posters handed out to all the nurses, surgeons, anaesthetists (Sx06AB-T-short) | - We were informed by letters (Ax08NB-L-short) - Information posted demonstrating some of the things that had been discovered on account of the surgical checklist (Ax07BC-L-short) |
| Discussion at staff or team meetings | - We have a staff meeting every Wednesday, basically that’s how we did it (Nx15BC-S-short) - I met with each department at their monthly meetings to explain the process (Nx02ON-L-long) | - Brought up at the monthly department of surgery meeting (Sx09ON-T-long) | - General discussion between nurses and administration (Ax08NB-L-short) - Number of meetings of the Surgical Services Committee (Ax05BC-L-short) |
| Education session or presentation | - A training session for the physician group and then a training session for the nurses (Nx20SK-S-long) - We had a power point presentation on how the checklist worked (Nx22AB-T-long) - We did case scenarios (Nx26ON-T-short) - We did a dry run of how we would expect it to be used (Nx18AB-S-short) - A link to the web video was available in our staff room (Nx20SK-S-long) - we made our own video (Nx22AB-T-long) | - Rounds with anaesthesia, nursing and surgery (Sx06AB-T-short) - Nurses had several in services (Sx08BC-T-short) - We had a presentation at rounds (Sx13ON-L-long) | - One presentation to the Department of Surgery, one presentation to the Department of Anaesthesia (Ax05BC-L-short) |
| Promotional reminders in the operating room | - Safe surgery OR caps for the nurses (Nx10AB-S-short) - It was a towel that covered all the instruments and it was orange and alerted them that we are not going ahead until we do the checklist (Nx19QC-S-short) | --- | --- |
| Facilitator or champion | - Champions helped us spearhead how we were going to discuss this (Nx01NB-L-short) - The educator was in the rooms initially to help troubleshoot (Nx29AB-T-long) | - OR nursing educator was the main overall champion (Sx11ON-T-long) - One of the nurse educators was the champion (Sx13ON-L-long) | - In the first week of two a number of people were acting as observers and facilitators (Ax07BC) |
| Pilot test | - We had a pilot where we trialed it with one anaesthetist and one surgeon so that they could give feedback (Nx10AB-S-short) - We did a pilot for a month (Nx11BC-S-short) - We did a two week trial (Nx14ON-S-short) - We did a pilot project with one group (Nx05NS-L-short) | - We started with services that had complex patients where we felt it was most important that there be communication (Sx11ON-T-long) - It started with briefing and debriefing and then we incorporated other parts of the checklist (Sx12BC-T-long) | - An initial trial period for three months (Ax03QC-S-long) - It started in one particular surgical sub-specialty, ironed out the details, and then just kept going so it was a gradual thing (Ax04NB-T-long) |
| Collecting and sharing local data about use and impact | - We started posting the results on bulletin boards in the operating room by physician and by service and that created quite a bit of competition. We would communicate those good catches (Nx09ON-S-short) | --- | --- |
| Nurses responsible for planning and implementation | - A number of nursing staff from across the facility (Nx04PEI-L-long) - It is mostly nursing driven (Nx04BC-S-long) | - Clinical nurse leaders in the operating room (Sx08BC-T-short) - One of the nurse educators for the OR was the champion (Sx13ON-L-long) | - The head nurse for the operating theatre (Ax06NB-T-long) |
| Poorly implemented (imposed on staff rather than engaging them) | - We’ve heard “you’re not going to tell me how to practice medicine” (Nx22AB-T-long) - Our CEO basically said this has to be done (Nx06NS-L-short) | - It was never explained why they must be there or given the opportunity for some flexibility (Sx04BC-T-long) - It came from management level (Sx10ON-L-short) | - It’s been forced upon us. It’s hurt morale and caused tension in the operating room team (Ax05BC-L-short) - It came from above, it was just imposed (Ax09NB-T-long) |
| Lack of leadership from hospital | - Senior leadership. I just didn’t get the support that I would have needed to be able to implement that fully (Nx02ON-L-long) - If it’s not endorsed by leadership then it may not be as persuasive as it could be (Nx04PEI-L-long) - More involvement from senior leadership (Nx02ON-L-long) | --- | - Needs to be supported by the medical quality committee of the hospital (Ax02ON-L-short) |

| Time needed to pilot before full launch | - Take the time to introduce it as a trial (Nx21BC-S-short) - We would probably start one service at a time because it was extremely overwhelming to implement it all at the same time in five services and three theatres (Nx20SK-S-long) | --- | --- |
| --- | --- | --- | --- |
| Engage staff in planning and implementation | - It’s important to get them involved at the very beginning (Nx04PEI-L-long) | - Involve the people who’re gonna be using it in its development so you feel an ownership (Sx05BC-S-short) | - Involvement of the people performing the checklists in its development and implementation would certainly improve buy in (Ax07BC-L-short) |
| Support from external groups | - Have the support of your provincial ministry (Nx03AB-L-long) - Some of the bigger associations should have been involved before this even got considered to be implemented (Nx28AB-T-long) - The medical association and the college of physicians and surgeons don’t seem to have been engaged (Nx28AB-T-long) | --- | - Endorsement by organizations like the Canadian Medical Protective association, the Canadian Society of Anaesthesiologists, Canadian association of surgeons gives it a lot more weight (Ax03QC-S-long) |

Profession (Nx=nurse, Sx=surgeon, Ax=anesthetist), Province (two-letter identifier), Hospital type (S=small, L=large, T=teaching), Time using the surgical checklist (short=≤12 months, long=13+ months)

**Integration of the surgical safety** checklist

| Theme | Exemplary quotes from participants | | |
| --- | --- | --- | --- |
|  | Nurses | Surgeons | Anaesthetists |
| Introduced in OR as card, sheet or poster | - We laminated the checklist and put one in each OR (Nx03AB-L-long) - The checklist was enlarged and we put it up on the wall (Nx12QC-S-short) | - Laminated sheets (Sx11ON-T-long) - We have a large poster in each of the ORs (Sx08BC-T-short) | - Small cards were placed in every operating room (Ax07BC-L-short) - The checklist is posted on each of the OR walls (Ax02ON-L-short) |
| Documentation processes varied | Manual/Paper record   - The nurse will write on the record that the surgical checklist has been completed (Nx27QC-T-short) - Nurses in the OR have a booklet where they mark off for every case if the surgical checklist was used (Nx10AB-S-short)   Electronic/computer record   - It’s monitored in our electronic medical records (Nx02ON-L-long) | Electronic/Computer record   - It’s recorded electronically as to whether or not the checklist has been done (Sx09ON-T-long) | Manual/Paper record   - There is a tick box on one of the OR nursing forms (Ax07BC-L-short)   Electronic/Computer record   - Nurses have three check boxes on the electronic patient record (Ax01ON-T-long) |
| Resistance from medical staff | - There is lingering resentment on the part of some of the surgeons (Nx06NS-L-short) - Our bad actors are still there. Those surgeons who don’t want to do it (Nx24ON-T-short) - Getting buy-in from the surgeons and anaesthetists (Nx07NS-L-short) | - Surgeons were refusing to do it or not doing it well (Sx04BC-T-long) - Surgeons, nursing and anaesthesia didn’t think this was useful and were obstructive (Sx09ON-T-long) - Lingering beliefs in why it is necessary (Sx07AB-L-short) | - Surgeons were screaming that this is stupid (Ax01ON-T-long) - Surgeons were frustrated. Some of the anaesthesiologists didn’t want to be involved in this (Ax02ON-L-short) - Getting acceptance from surgeons and anaesthesiologists (Ax07BC-L-short) |
| Team members absent or not paying attention | - People who aren’t listening, aren’t participating, rolling their eyes (Nx01NB-L-short) - Everybody being in the room at the same time was one of our biggest challenges (Nx09ON-S-short) - Surgeons said it doesn’t actually say we have to be in the room (Nx14BC-S-short) - If you’re not careful it can become just one more tick box (Nx13SK-S-long) - If it’s not used properly the people that don’t engage in the checklist may get sloppy (Nx01AB-T-long) | - Everyone is talking so no one’s listening (Sx01ON-T-short) - A lot of surgeons aren’t there when the case is starting (Sx04BC-T-long) - I sometimes feel like people are just yes’ing through the checklist (Sx10ON-L-short) - Sometimes when you’re doing the checklist people aren’t even listening (Sx11ON-T-long) - There are a lot of people that are just going through the motions and are not really engaged (Sx12BC-T-long) | - If the surgeon is not paying attention nobody else is obligated to pay attention (Ax08NB-L-short) - Some of our surgeons leave the OR. Some days it doesn’t seem like a team effort at all (Ax09NB-T-long) |
| Time constraints, redundant or irrelevant | - When we’re doing the rapid turnover how do we navigate that (Nx29AB-T-long) - After hours and call backs and emergencies – it’s not used as well as it could be (Nx11BC-S-long) | - It does delay getting the case started (Sx04BC-T-long) - Parts of the checklist are not really relevant so people don’t see the value (Sx11ON-T-long) | - The perception of the redundance is huge. It’s an additional layer that doesn’t improve efficiency. It’s more time consuming (Ax07BC-L-short) |
| Nurses responsible for SSC review and documentation | - It’s a whole lot of documentation (Nx27QC-T-short) - It’s one more job for the circulating nurse to do (Nx02ON-L-long) | - Nurses initiate the process. They have a card that they read off and they also have to mark that the safety check was done (Sx02ON-S-short) - Documentation has dramatically increased, and it has taken away from patient care (Sx09ON-T-long) | - The nurses, they perform the checklist for every case (Ax08NB-L-short) - Layering on further administrative burden (Ax07BC-L-short) |
| Unclear who is responsible or how to use it | - People not fully understanding what the components of the checklist were (Nx25ON-T-long) - We had to remind nurses that we have to make sure it’s initiated (Nx14ON-S-short) | - I’m still unclear as to who it is that’s supposed to lead the discussion (Sx04BC-T-long) - We don’t know how to do it. We’re not trained (Sx12BC-T-long) | - Could have done a better job of educating people as to who’s role was what (Ax04NB-T-short) |
| Hierarchical culture of operating room | - Some nurses are really shy to speak up (Nx26ON-T-short) - We had to adjust the OR culture to reinforce that we don’t believe in hierarchy (Nx20SK-S-long) | - Flatten the hierarchy (Sx12BC-T-long) | - Nurses were intimidated so there was friction between team members (Ax02ON-L-short) |
| Evidence of impact from research needed | - Hard data – people like to know that this is evidence-based (Nx29AB-T-long) | - Ongoing data demonstrating its impact on patient care (Sx09ON-T-long) - We need more studies that are of better quality (Sx12BC-T-long) | - Continuing literature produced as we gather more information to determine how relevant it is to our medical practice here (Ax06NB-T-long) |
| Local data on use and impact needed | - Sharing the information that we find about good catches (Nx24ON-T-short) | - Feedback about compliance compared to before the checklist was implemented (Sx08BC-T-short) | - Feedback that was accurate and meaningful (Ax07BC-L-short) |
| Physician champions needed | - Need real leaders in the physician group to help sell it (Nx23ON-T-long) - I would push that it be physician led (Nx28AB-T-long) | - A surgeon champion that everyone knows (Sx04BC-T-long) - A surgeon stands there and articulates it (Sx07AB-L-short) | - A facilitator to help people in going through the process (Ax04NB-T-short) |
| Networking with, and updates from other hospitals may identify useful strategies for integration | - Networking with other facilities (Nx11BC-S-long) - Updates of how people are modifying their checklist (Nx26ON-T-short) - To check in with each other’s experiences (Nx28AB-T-long) | --- | --- |
| Continuing education needed | - Re-invest in checklist education on a regular basis (Nx22AB-T-long) - Recognize that individual and team learning is ongoing (Nx04PEI-L-long) | - Formal orientation for newcomers (Sx04BC-T-long) - A training program (Sx12BC-T-long) - Formal orientation for newcomers (Sx04BC-T-long) | - More training and education (Ax08NB-L-short) |

Profession (Nx=nurse, Sx=surgeon, Ax=anesthetist), Province (two-letter identifier), Hospital type (S=small, L=large, T=teaching), Time using the surgical checklist (short=≤12 months, long=13+ months)

**Monitoring of surgical safety checklist adherence**

| Theme | Exemplary quotes from participants | | |
| --- | --- | --- | --- |
|  | Nurses | Surgeons | Anaesthetists |
| Observation or spot checks | --- | - The nursing educator will go around occasionally to see what the quality is but that can be variable (Sx11ON-T-long) | - Several members of the OR management team would go from room to room and observe if it was being followed (Ax07BC-L-short) - They’ve actually had people that were anonymous audit the compliance (Ax09NB-T-long) |
| Not recorded or unsure | - I don’t think we’re documenting if it was done (Nx04PEI-L-long) | - Nobody’s writing it down. It’s verbal (Sx10ON-L-short) | - I don’t believe it is recorded to my knowledge (Ax07BC-L-short) |
| Compliance audits or reports | - I gather a copy of it every OR day, and see if there’s any concerns or problems (Nx18AB-S-short) - We do audits quarterly to see what our compliance is (Nx01NB-L-short) | - An audit is done on a sporadic basis (Sx06AB-T-short) - It’s posted on a monthly basis (Sx09ON-T-long) | - We have routine audits done every three months (Ax05BC-L-short) |
| Communication of reports | Staff   - We report them out to people weekly (Nx24ON-T-short)   Hospital managers   - There is a monthly report that gets sent to the manager (Nx14ON-S-short)   Government   - We mail it in once a week to [government] (Nx15BC-S-short) | Staff   - A report is forward to our Quality Committee and it’s posted on our surgical services score card on the intranet (Sx09ON-T-long)   Hospital managers   - The department head monitors this with administration of the hospital (Sx07AB-L-short) | Staff   - It’s posted on a board demonstrating some of the things that have been discovered on account of the surgical checklist (Ax07BC-L-short) |
| Addressing non-compliance | Nurse manager speaks to individual   - I would try and talk with the OR crew and the docs to find out what happened and why (Nx18AB-S-short) - I follow up with them individually (Nx12ON-S-short)   Incident report   - An incident report would be made out (Nx04PEI-L-long)   Considered by team/committee   - The team would analyze it and decide on appropriate action (Nx28AB-T-long)   Reported to management   - Reported to the chief of medical staff and he addresses it (Nx10AB-S-short) | Incident report   - An incident report would be filled out (Sx09ON-T-long)   Considering by team/committee   - It would be identified mainly by the nurses that it didn’t happen then we try to review it in the OR committee monthly (Sx06AB-T-short)   Reported to management   - It would be escalated up to the clinical director or the chief of surgery to come and deal with it (Sx02ON-S-short) | Nurse manager speaks to individual   - They go to the head nurse of the operating room for review and she would question the people that were involved (Ax06NB-T-long)   Incident report   - We have a compliance report that comes out every month in which issues are noted and then those are addressed by the team who was involved (Ax02ON-L-short)   Reported to management   - The OR nurse would come and speak to me. I would speak to the Chief of Surgery and then they would speak to the individual surgeon (Ax01ON-T-long) |
| Consequences | - The CEO said that surgeons that weren’t compliant would lose their privileges (Nx15BC-S-short) | --- | --- |
| No consequences | - There were no consequences if somebody was acting up (Nx02ON-L-long) | - There’s no punishment (Sx07AB-L-short) | - I’m not sure if there would be feedback (Ax09NB-T-long) |
| Mandatory documentation and reporting, and associated consequences | - If it was mandatory that we had to report people might be more apt to be more diligent (Nx05NS-L-short) - If there was a consequence (Nx02ON-L-long) | --- | - Consequences for it not being done properly (Ax04NB-T-short) - Buy in is mandatory and if you don’t participate you may lose your privileges (Ax09NB-T-long) |
| Less documentation | - Clerical support for making the changes to the form and setting up data collection (Nx06NS-L-short) | - We have to make sure we don’t turn people off with too much documentation (Sx11ON-T-long) | --- |
| Ongoing review or audit | - Audits to make sure that everything is happening (Nx28AB-T-long) | - Continued audits to figure out what areas we’re not compliant with and figure out strategies to change that (Sx08BC-T-short) | - Checking to see if the checklist is actually being done (Ax04NB-T-short) - Follow up to see if improvements in the system had actually been carried out (Ax06NB-T-long) |
| Auditing by external groups | - People coming from the ministry to do those audits (Nx02ON-L-long) | - Random audits by someone who is not actually part of the operating team (Sx11ON-T-long) | --- |

Profession (Nx=nurse, Sx=surgeon, Ax=anesthetist), Province (two-letter identifier), Hospital type (S=small, L=large, T=teaching), Time using the surgical checklist (short=≤12 months, long=13+ months)
